# Adjuvant Intravitreal Bevacizumab for Retinal Neovascularization in Eales’ Disease Associated With Latent Mycobacterium Tuberculosis

**DOI:** 10.7759/cureus.41003

**Published:** 2023-06-26

**Authors:** Orlando G Gonzalez Martinez, Victor M Villegas, Armando L Oliver, Carmen Santos

**Affiliations:** 1 Ophthalmology, University of Puerto Rico School of Medicine, Medical Sciences Campus, San Juan, USA

**Keywords:** case report, photocoagulation, intravitreal bevacizumab, tuberculosis, eales’ disease

## Abstract

We report a case of bilateral Eales’ disease managed with intravitreal bevacizumab. A 32-year-old woman with a history of bacillus Calmette-Guerin vaccine, administered when she was 10 years old, presented with a five-day history of a scotoma in the temporal field of her right eye. A dilated fundus exam and fluorescein angiography showed bilateral retinal peripheral capillary non-perfusion, retinal neovascularization in the right eye, and deep intraretinal hemorrhages in the left eye. Her laboratory workup resulted in a positive QuantiFERON-TB Gold test (Cellestis Ltd, Carnegie, Victoria, Australia). Chest computed tomography showed a calcified granuloma in her right lung. Angiographic-guided pan-retinal photocoagulation was performed, and intravitreal injections of bevacizumab (1.25 mg/0.05 mL) were administered in both eyes over the course of three months. The intraretinal hemorrhages resolved after three months of therapy. Three months following treatment, the patient showed normal fundus findings without any evidence of recurrence and a visual acuity of 20/20 in both eyes. Intravitreal bevacizumab in combination with angiography-guided pan-retinal photocoagulation may be efficacious in select patients with Eales’ disease.

## Introduction

Eales’ disease is a rare idiopathic occlusive vasculitis characterized by retinal venous inflammation, vascular occlusion, and subsequent retinal neovascularization involving the mid-peripheral retina [1-4]. Most patients with the disease are men, and the condition is most prevalent in India and the Middle East [5,6]. While the etiology of Eales’ disease is not clearly defined in the literature, several studies have shown that the disease is associated with *Mycobacterium tuberculosis* (*M. tuberculosis*) [3,7,8]. In their 2021 study, Gupta and Biswas found, using the QuantiFERON-TB Gold assay (Cellestis Ltd, Carnegie, Victoria, Australia), that 56% of their patients, all with Eales’ disease, tested positive for *M. tuberculosis* [8]. The pathophysiology of Eales’ disease involves inflammation of the retinal vessel walls leading to retinal periphlebitis, ischemia, and neovascularization [4,7]. The etiology of Eales’ disease remains poorly understood; however, several clinical studies have proposed an etiology involving an immunological reaction in response to an exogenous agent [4,7]. Tuberculosis exposure and hypersensitivity to tuberculoprotein have been suggested as potential factors associated with the development of this disease [4,5,7]. Without adequate treatment, a vitreous hemorrhage and tractional retinal detachment may develop [4]. The treatment for this disease may include pan-retinal photocoagulation, systemic corticosteroid therapy, antitubercular therapy, and, most recently, the intravitreal administration of the anti-vascular endothelial growth agents, such as bevacizumab [4,8]. Therefore, we report herein on the treatment of a case of bilateral Eales’ disease associated with latent *M. tuberculosis.*

## Case presentation

A 32-year-old woman from Romania with no previous history of medical or ocular disease presented with a five-day history of a scotoma in the temporal field of her right eye (OD). She had no history of pain, photophobia, discharge, ocular surgery, or trauma. Her past medical history was remarkable for a previous bacillus Calmette-Guerin vaccine, which she received when she was 10 years old. The family history was noncontributory. Her recent travel included having lived in India for the two years prior to her visit to our office.

Upon a comprehensive ophthalmic evaluation, her best-corrected visual acuity at the initial presentation was 20/20 in both eyes (OU). Intraocular pressures were within normal limits, bilaterally. The patient’s pupils were equally round and reactive to light. The anterior segment was unremarkable OU. No evidence of anterior segment flare or inflammatory cells was present. She had 1+ vitreous cells, and there was no evidence of vitreous haze OU.

**Figure 1:**
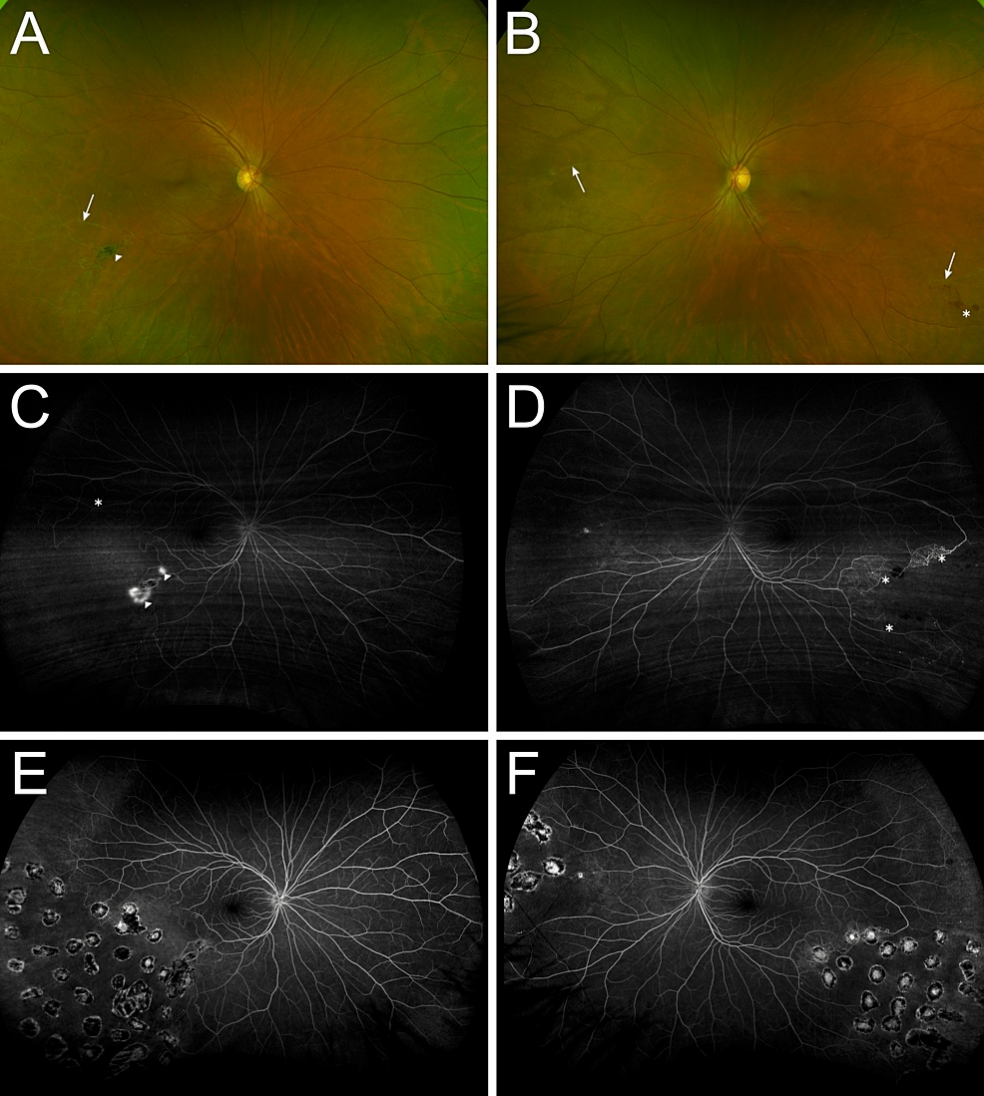
Ultra-widefield fundus imaging of the right and left eyes at presentation. (A) Color photography of the right eye reveals peripheral sheathed vessels (arrow) and an area of retinal neovascularization (arrowhead) along the inferotemporal arcade. (B) Color photography of the left eye reveals sheathed vessels (arrows) and deep intraretinal hemorrhages (asterisk). (C) Fluorescein angiography of the right eye reveals peripheral capillary non-perfusion (asterisk) corresponding with the areas of vascular sheathing and an area of leakage corresponding to the site of retinal neovascularization (arrowheads). (D) Fluorescein angiography of the left eye reveals peripheral capillary non-perfusion (asterisks) corresponding to the areas of vascular sheathing. Fluorescein angiogram of the right (E) and left (F) eyes three months after treatment reveals resolution of the retinal neovascularization in the right eye, resolution of the hemorrhages in the left eye, and no progression of the areas of non-perfusion.

A dilated fundus examination showed sheathed vessels within the temporal periphery OU and the nasal periphery of her left eye (OS), deep intraretinal hemorrhages OS, and an area of retinal neovascularization within the inferotemporal arcade OD (Figures 1A-1B). A fluorescein angiogram revealed peripheral capillary non-perfusion corresponding to the areas of retinal arteriolar occlusion OU and leakage within the area of retinal neovascularization OD (Figures 1C-1D). Spectral-domain optical coherence tomography did not reveal any signs of macular edema OU. Figures 1E-1F show resolution after treatment.

A complete laboratory workup was performed. Her QuantiFERON-TB Gold Plus test was positive for *M. tuberculosis*. Chest computed tomography revealed a calcified granuloma in her right lung. Brain magnetic resonance imaging with and without intravenous gadolinium was unremarkable. Histocompatibility leukocyte antigen (HLA) testing was negative for HLA-A29 and positive for HLA-B51. Workup, including HIV, syphilis, hepatitis, *Bartonella* antibodies, fluorescent treponemal antibody absorption, antineutrophil cytoplasmic antibody, antinuclear antibody (ANA), and angiotensin-converting enzyme levels (ACE), was negative.

A presumptive diagnosis of Eales’ disease associated with latent tuberculosis was made. The patient was seen by a pneumologist and started on antitubercular therapy. The patient underwent angiographic-guided pan-retinal photocoagulation in areas of non-perfusion OU. Bilateral intravitreal injections of bevacizumab (1.25 mg/0.05 mL) were given every six weeks for three months. At the three-month follow-up examination, the patient was asymptomatic, with a visual acuity of 20/20 OU. A fluorescein angiogram showed resolution of the retinal neovascularization OD, resolution of the hemorrhages OS, and no progression of the areas of non-perfusion OU (Figures 1E-1F).

## Discussion

The etiology of Eales’ disease is still poorly understood. Studies suggest that the disease process is linked to* M. tuberculosis* exposure, particularly to the tuberculoprotein and tubercle bacilli [3,8]. Other factors and immunologic mechanisms involving oxidative stress, vitamin C and E reduction, and increased vascular endothelial growth factor have been proposed, but the exact pathophysiological mechanism is still unknown [9]. The fact that this disease seems to be linked to ocular inflammation and hypersensitivity to tuberculin proteins suggests that there may be immunologic mechanisms that are yet to be understood [4,9].

Eales’ disease progresses in stages, with the initial inflammatory phase showing periphlebitis, venous dilation, and perivascular exudates [4,7]. The intermediate stage is characterized by capillary ischemia and the demarcation of perfused and non-perfused zones by veno-venous shunts, venous beading, and microaneurysms [4,7]. The final, proliferative stage involves neovascularization occurring at the junction between the perfused and non-perfused areas of the retina, leading to recurrent vitreous hemorrhages with or without retinal detachment [4,7]. Fluorescein angiography may show leakage from the sheathed vessels, retinal vascular tortuosity, and/or telangiectasia, shunt vessels, venous stasis, capillary non-perfusion, and retinal neovascularization [4,7].

A diagnosis of Eales’ disease is a diagnosis of presumption and exclusion, as there are no laboratory tests that are specific or sensitive for the diagnosis of Eales’ disease [4,7,10]. In the absence of other systemic conditions, peripheral retinal inflammation and recurrent vitreous hemorrhages are important defining features [4,7]. A chest X-ray, a Mantoux tuberculin skin test, and an interferon-gamma release assay test may be useful, given the disease’s association with the genetic material of *M. tuberculosis *in the vitreous of affected patients [11]. A Venereal Disease Research Laboratory (VDRL) test is useful to assess for syphilis, as is an ANA test for systemic lupus erythematosus [12]. Hemoglobin electrophoresis can assess for sickle cell retinopathy. An ACE test and a chest X-ray can test for sarcoidosis [12]. In this case, the positive presence of HLA B-51 without a history of genital or oral ulcers renders Behçet’s disease an unlikely diagnosis [13].

The treatment of Eales’ disease should be based on disease severity and should aim to prevent deterioration and complications, such as retinal neovascularization, vitreous hemorrhage, and retinal detachment. Historically, patients have undergone pan-retinal photocoagulation in areas of non-perfusion [4,7]. Other reported treatments have included oral corticosteroids and systemic antitubercular therapy [2,4,7]. In cases of non-clearing vitreous hemorrhage or tractional retinal detachment threatening the macula, surgical management with vitrectomy is indicated [4]. Recently, anti-vascular endothelial growth factor agents have been reported to be effective in lessening the need for pan-retinal photocoagulation in patients in the early stages of Eales’ disease [14]. Similar to what was seen in our patient, other patients who were treated with intravitreal bevacizumab and peripheral photocoagulation in ischemic areas achieved the stabilization of their disease without any signs of recurrence [15,16]. Intravitreal bevacizumab may become an essential part of treatment in patients with recurrent vitreous hemorrhage that prevents the administration of peripheral photocoagulation. Reported cases have shown that once bevacizumab is administered, vitreous hemorrhages stop occurring, allowing for photocoagulation [16]. Some case studies have shown the rapid regression of disc and retinal neovascularization following the use of intravitreal bevacizumab in patients with Eales’ disease, even in those who showed a negligible response to laser therapy [17].

In our case, the early diagnosis and treatment of this patient most likely prevented the manifestation of the classic sequelae of untreated Eales’ disease, which involves recurrent vitreous hemorrhages but also includes additional neovascularization, retinal detachment, epiretinal membrane formation, neovascular glaucoma, and cataracts. Early treatment may help reduce the need for vitrectomies in the future for this patient and improve her visual prognosis, as long as she continues to do the appropriate follow-up.

## Conclusions

Intravitreal bevacizumab in combination with angiography-guided photocoagulation of ischemic areas may be efficacious in select patients with Eales’ disease. The use of ultra-widefield fundus photography and ultra-widefield fluorescein angiography may aid in the diagnosis and treatment of this condition. Further studies should be conducted to confirm the long-term efficacy and safety of this treatment modality.
